# Dolphin Morbillivirus in a Cuvier’s Beaked Whale (*Ziphius cavirostris*), Italy

**DOI:** 10.3389/fmicb.2017.00111

**Published:** 2017-01-31

**Authors:** Cinzia Centelleghe, Giorgia Beffagna, Giuseppe Palmisano, Giovanni Franzo, Cristina Casalone, Alessandra Pautasso, Federica Giorda, Fabio Di Nocera, Doriana Iaccarino, Mario Santoro, Giovanni Di Guardo, Sandro Mazzariol

**Affiliations:** ^1^Department of Comparative Biomedicine and Food Science, University of PadovaLegnaro, Italy; ^2^Department of Animal Medicine, Production and Health, University of PaduaLegnaro, Italy; ^3^Istituto Zooprofilattico Sperimentale del Piemonte, Liguria e Valle d’AostaTorino, Italy; ^4^Istituto Zooprofilattico Sperimentale del MezzogiornoPortici, Italy; ^5^Faculty of Veterinary Medicine, University of TeramoTeramo, Italy

**Keywords:** dolphin morbillivirus, cross-species infection, Cuvier’s beaked whale, virology, Mediterranean Sea

## Abstract

Dolphin morbillivirus (DMV) has caused several mortality events in Mediterranean striped (*Stenella coeruleoalba*) and bottlenose (*Tursiops truncatus*) dolphins populations since 19; in the last 5 years, the virus was reported to infect new hosts in this basin, such as fin whales (*Balaenoptera physalus*), sperm whales (*Physeter macrocephalus*), and even a harbor seal (*Phoca vitulina*). Very recently, a calf Cuvier’s beaked whale (*Ziphius cavirostris*) calf stranded on the Southern Italian coastline with mild pathological findings suggestive of morbilliviral infection, received the first confirmation of DMV infection in this species by biomolecular evidences on lung tissue. This new cross-species infection report, along with 19% of the cetaceans specimens examined by the Italian Stranding Network being found positive to DMV, support the hypothesis of an endemic circulation of this virus among Mediterranean cetaceans.

## Background

*Cetacean morbillivirus* (CeMV), belonging to genus Morbillivirus, has caused several mortality events worldwide over the past 30 years. Besides its genomic and antigenic relationship with morbilliviruses infecting *Cetartiodactyla*, namely Rinderpest and Peste des Petits Ruminants Viruses (RPV and PPRV; Van Bressem et al., 2014), CeMV, similarly to Canine Distemper Virus (CDV) has also the capacity to infect several species belonging to different orders, particularly in the Mediterranean Sea (Van Bressem et al., 2014; De Vries et al., 2015). In this basin, the dolphin morbillivirus (DMV) strain has been responsible for at least four outbreaks reported in 1990–1992, in 2006–2008, in 2011, and in 2013, respectively, and mainly affecting striped dolphins (*Stenella coeruleoalba*), bottlenose dolphins (*Tursiops truncatus*), and long-finned pilot whales (*Globicephala melas*; Van Bressem et al., 2014). In these species, typical pathological findings related to an acute DMV infection are a severe non-suppurative meningo-encephalitis, a necrotizing bronco-interstitial pneumonia with evidence of secondary infections and presence of multinucleated syncytia and, even more relevant, lymphoid cell depletion with subsequent immune system impairment (Van Bressem et al., 2014; Di Guardo and Mazzariol, 2016).

*Delphinidae* seem to be particularly susceptible to DMV infection due to the molecular affinity existing between the agent and their SLAM/CD150 viral receptor molecule (Sato et al., 2012). Notably, in the latter two Mediterranean outbreaks, an apparent expansion of the DMV host range has been observed, along with the occurrence of consistently milder pathological changes in affected cetaceans involving only lymphoid tissues (Casalone et al., 2014). More in detail, biomolecular (reverse transcription polymerase chain reaction, RT-PCR) and/or immunohistochemical (IHC) evidence of DMV infection has been reported in fin whales (*Balaenoptera physalus*; Mazzariol et al., 2016), sperm whales (*Physeter macrocephalus*; West et al., 2015; Centelleghe et al., 2016), and in a captive harbor seal (*Phoca vitulina*; Mazzariol et al., 2013). Furthermore, in 2015, thanks to a constant monitoring effort put in place by the newborn Italian Stranding Network, DMV was molecularly detected in no less than 19% of 56 stranded delphinids, namely in five bottlenose dolphins, five striped dolphins, and one Risso’s dolphin (*Grampus griseus*), sometimes showing typical *post-mortem* findings related to morbilliviral infection (Italian Diagnostic Report on Stranded Cetaceans, 2015).

## Case Presentation

In November 2015, a male Cuvier’s beaked whale (*Ziphius cavirostris*) calf was found stranded alive along the Tyrrhenian coast of Calabria. Few hours afterward the animal died and a complete field necropsy was carried out within 24 h from death. According to standard protocols, tissues were routinely collected from the aforementioned animal for histological and microbiological examinations. Due to field conditions, which did not allow an immediate samples’ freezing, each tissue was collected separately at room temperature in RNA-later preserved solution (Life Technologies) during the necropsy and subsequently transported and frozen approximately within 72 h after death.

Total RNA extraction was performed by pressure filtration method, using PureLink RNA Mini Kit (Ambion, Thermo Scientific) following manufacturer’s instructions and the six micrograms of the RNA obtained were used for the reverse transcriptase reaction. Reverse transcriptase reaction, performed with PureLink RNA Mini Kit (Ambion, Thermo Scientific) following manufacturer’s instructions, was carried out employing a previously published protocol using a primer named DMV2 (Belliere et al., 2011).

At first, in order to detect DMV RNA, a recently described nested PCR technique targeting an highly preserved region of the viral H gene, was used (Centelleghe et al., 2016). Thereafter, viral cDNA amplification was performed using previously reported (Belliere et al., 2011) and newly designed overlapping primer pairs (**Table 1**) with Phusion Hot Start II DNA Polymerase (Thermo Scientific) and at the following PCR conditions: 30 s at 98°C; 35 cycles of 10 s at 98°C, 30 s at 56°C, 1 min at 72°C; and 10 min at 72°C. Next, the DNA fragments obtained were purified, cloned into the plasmid vector pCR-Blunt II-TOPO (Invitrogen, Life Technologies, Carlsbad, CA, USA) according to the manufacturer’s instructions, and finally sequenced. In order to edit, assemble, and translate sequences, programs in the DNASTAR Lasergene software package^1^ were used. A phylogenetic tree based on a collection of DMV and PWMV partial sequences of the P gene was reconstructed using PhyML.

**Table 1:** Primer sequences used in nested reverse transcription polymerase chain reaction (RT-PCR) protocol and conventional RT-PCR protocol.^∗^

Primer name (Reference)	*nt* position (referred to AJ608288)	5′→3′ sequence (sense)	Fragment length, bp	Annealing temperature
DMV-2 (Belliere et al., 2011)	15702–15684	ATHCCCAGCTTTGTCTGGT	cDNA production	
DMV-11F (Mazzariol et al., 2016)	7799–7819	CCGAACCTGATGATCCATTT	612	56°C
DMV-11R (Mazzariol et al., 2016)	8411–8391	CGTAAATGTCCATCCCTGCT		
DMV-13F (Centelleghe et al., 2016)	8052–8072	CATCATAGGGGGTGGTTTGA	200	62°C
DMV-13R (Centelleghe et al., 2016)	8232–8212	GGGGTGGTCTACTCTTGCAC		
DMV-1F (This study)	72–92	TCAATTGGCACAGGATTTGG	474	56°C
DMV-1R (This study)	545–525	CCAATGGGTTCCTCTGGTGT		
DMV-2F (This study)	501–521	TCTATTCAAGCAGGGGAGGA	622	56°C
DMV-2R (This study)	1122–1102	TCGGCTGTGATCCCTAGTTC		
DMV-N1 (Belliere et al., 2011)	1203–1222	CAAGAGATGGTCAGGAGATC	1358	56°C
DMV-P2 (Belliere et al., 2011)	2541–2521	GACAGGTGGTGCAACCCGAC		
DMV-C (Belliere et al., 2011)	2132–2152	ATGTTTATGATCACGGCGGT	769	56°C
DMV-4R (This study)	2900–2880	AGGTGGCCTTCGATAGTTGA		
DMV-5F (This study)	2439–2459	ACCAATTCCAACCTCAGTGC	716	56°C
DMV-5R (This study)	3154–3134	ATCCCACAGCAGAGCTCATT		
DMV-14F (This study)	3037–3057	CCAGCAGTCGAGAGAAATCC	723	56°C
DMV-14R (This study)	3759–3739	TCTCATTTAACCCCGCTGTC		

*^∗^nt, nucleotide; bp, base pair.*

**FIGURE 1:**
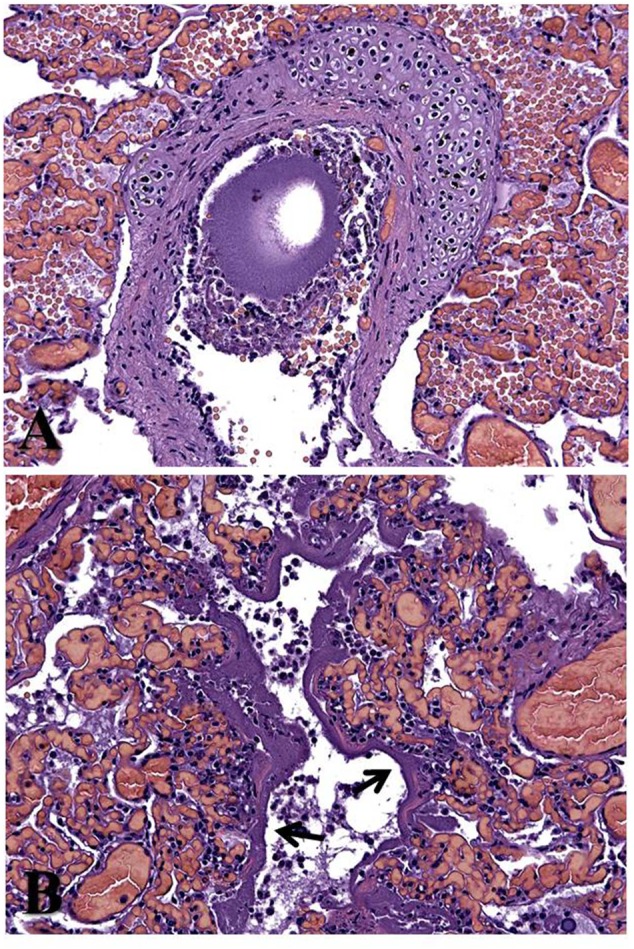
**Haematoxylin and eosin-stained lung tissue section from Cuvier’s beaked whale (*Ziphius cavirostris*) calf stranded off the Southern Italian coastline.** Fibrinous bronco-pneumonia, associated with necrotizing bronchiolitis. **(A)** Necrotic cellular debris is shown within the airways. **(B)** Depots of fibrinous membranes (hyaline membrane, arrows) are seen covering the alveolar epithelium, along with a mild neutrophilic infiltration. Original magnification × 20.

With the exception of a poor body condition, no relevant gross findings were seen, while microscopic investigation revealed hyaline membrane deposits along the lung alveolar walls, with necrotic debris being also found in airways *lumina* and with a final morphologic diagnosis of a multifocal, fibrinous bronco-pneumonia with mild, multifocal, necrotizing bronchiolitis. Furthermore, haemorrhagic foci and pulmonary congestion were observed (**Figure 1**). Microbiological investigations allowed recovery of verocytotoxic (VT1) *Escherichia coli* (Borriello et al., 2012) from the aforementioned pulmonary lesions.

Dolphin morbillivirus RNA was detected in RNA-later-preserved lung tissue by means of the aforementioned nested PCR technique targeting the viral H gene, encoding the haemagglutinin (H) antigen (Centelleghe et al., 2016; GenBank provisional Acc. No. KX237512). The gel electrophoresis showed a result of good quality for both PCR steps of nested PCR (**Supplementary Figure 1**), probably due to the fact that tissues used for RNA extraction was of satisfactory quality: tissues, in fact, were collected within 24 h after death.

Conventional RT-PCR analysis allowed also to detect and sequence the complete N and P/V/C genes, encoding, respectively, for nucleocapsid (N) protein and phosphoprotein (P) antigens (provisional Acc. No. KX237510 for N gene and KX237511 for P/V/C gene).

These fragments, from lung sample, showed 99.11% sequence homology for the complete N gene, 99.34% sequence homology for the complete P/V/C gene, and 99.51% sequence homology for the partial H gene within the complete DMV genome (GenBank Acc. No. AJ608288). Furthermore, the P gene sequence found in the beaked whale herein investigated showed 99.05% homology with a CeMV isolate characterized from seven Longman’s beaked whales (*Indopacetus pacificus*) during a mass stranding in the South Pacific Ocean (GenBank Acc. No. KR704575; Garrigue et al., 2016).

**FIGURE 2:**
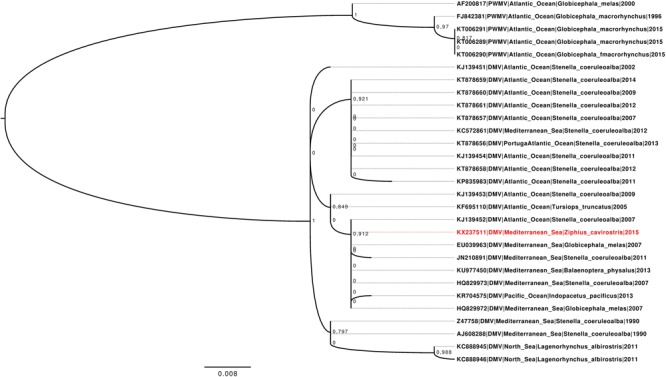
**Maximum likelihood phylogenetic tree reconstructed based on a collection of dolphin morbillivirus (DMV) and PWMV (used as outgroup) partial (285 nucleotides) sequences of the P gene.** The robustness of each clade *monophyly* has been evaluated using a fast non-parametric version of the aLRT (Shimodaira–Hasegawa [SH]-aLRT), developed and implemented in the PhyML3.0 (values are displayed near to the corresponding node). The strain reported in the present study has been showed in red.

The close relationship with other DMV detected in different cetacean species in both Mediterranean Sea and Atlantic and Pacific Oceans after 2007 was confirmed by nucleotide sequences alignment (**Supplementary Figure 2**) and phylogenetic analysis (**Figure 2**).

## Discussion

Until now, DMV infection has not been described as a pathological condition affecting *Ziphidae*, with the only exception in such context being represented by Beaked Whale Morbillivirus (BWMV), a CeMV strain infecting these species, which has been reported only in the Pacific Ocean (West et al., 2013; Jacob et al., 2016). In the Mediterranean Sea, beaked whales were never involved during previous DMV outbreaks (Van Bressem et al., 2014). In this respect, the homology with the CeMV isolate detected in Longman’s beaked whales as well with the morbillivirus affecting Mediterranean striped dolphins during the 1990–1992 epidemic suggest a possible adaptation of DMV to different species. In fact, as previously stated, despite DMV is not closely related to CDV, it seems to show a similar host range-related behavior (De Vries et al., 2015), thus being able to infect odontocete and mysticete species (Van Bressem et al., 2014). The reports of “new” hosts could be due, at least in part, to the rare occurrence of some species and/or to bad preservation status in which cetacean carcasses are often found. Another hypothesis related to a true “jump” into other species as a result of an endemic DMV circulation in the Mediterranean basin. In this respect, the results of a recent diagnostic surveillance activity, performed by a number of Public Veterinary Institutions involved in the Italian Cetacean Stranding Network, confirm that 19% of cetaceans found stranded along the Italian coastline were molecularly positive for DMV in 2015, without any evidences of ongoing epidemic events (Italian Diagnostic Report on Stranded Cetaceans, 2015). These results support the hypothesis of an endemic presence and circulation of DMV among Mediterranean cetaceans, with simultaneous and/or subsequent occurrence of likely spill-over events, as already documented in the case of the aforementioned harbor seal (Mazzariol et al., 2013).

In contrast to host-specific pathogens belonging to the same genus, such as Measles Virus, RPV and PPRV, the broad and expanding host range of DMV, testified by the present report and by numerous cases of DMV infection reported in the last 5 years in the Mediterranean Sea (Mazzariol et al., 2013, 2016, 2017; Centelleghe et al., 2016), being additional corroborated by the results of recent sero-epidemiological surveys (Profeta et al., 2015), appears to show a number of similarity with the behavior of CDV, despite the low phylogenetic and genetic homology degree between these two morbilliviruses (Van Bressem et al., 2014). Spill-over events at the end epidemic outbreaks or during endemic infection’s condition, resulting from interactions between CDV-infected dogs and wild carnivore and pinniped species, have led to mass mortalities (Di Guardo et al., 2005; Beineke et al., 2015). Viral host-jumps similar to those observed for DMV was already report for CDV infection in Amour tiger (*Panthera tigris altaica*) and in Serengeti Park lion (*Panthera leo*) populations, probably caused by a spill-over of CDV from domestic dogs and associated with a severe decline in wild carnivores worldwide and mass dog vaccination (Seimon et al., 2013; Viana et al., 2015). DMV epidemiology depends upon several factors, such as agent’s virulence, population’s antiviral immunity, and susceptible population’s density (Beineke et al., 2015). The viral strains responsible for these strandings events in new hosts exhibit a marked genetic relatedness with those which caused mass die-offs in early nineties and between 2006 and 2008 (Van Bressem et al., 2014). This observation strongly supports a prolonged DMV circulation in the Western Mediterranean, on one side, coupled with the presence of an inadequate level of antiviral immunity in cetaceans inhabiting this area, on the other.

## Concluding Remarks

The progressively increasing number of aquatic mammals species reported to be susceptible to DMV infection, such as beaked whales, with Morbillivirus-related pathological findings and with mortality trends non-related to ongoing epidemics, argue in favour of an endemic circulation of DMV in the Western Mediterranean area, with associated occurrence of periodic and self-limiting disease outbreaks like those occurred in 2011 (Di Guardo et al., 2013) and in 2013 (Casalone et al., 2014).

The lower density of striped dolphins and bottlenose dolphins, considered to be classical DMV hosts (Van Bressem et al., 2014), reported by recent monitoring efforts in the Mediterranean basin (Panigada et al., 2011), could have played a relevant role in cross-species infection, along with other potential factors, thereby bringing DMV in close contact with alternative hosts. In fact, the rate and intensity of intra- and inter-specific contacts may be relevant as well as the evolutionary relatedness of the concerned species (Parrish et al., 2008).

Similarly to wild carnivores for CDV, the species recently reported to be susceptible to morbilliviral infection, such as that herein described, could subsequently act as reservoirs for DMV, thus being a possible source of spillback events involving naive population of classic hosts and supporting the presence and circulation of DMV in the Mediterranean area (Beineke et al., 2015; Mazzariol et al., 2016, 2017).

The herein investigated, DMV-infected beaked whale adds further support to the important role likely played by vertical transmission in the infection’s epidemiology, with special emphasis on the strategies developed by DMV to gain an efficient entry and survival inside “non-classical/non-canonical” cetacean host species. (West et al., 2015; Centelleghe et al., 2016; Mazzariol et al., 2016, 2017). Finally, the involvement of newborns in the epidemiology and ecology of DMV infection, associated with that of young and pregnant females, could represent an additional, serious threat for the conservation of this already endangered species.

## Ethics Statement

Tissues for this project have been provided by the Mediterranean Marine Mammal Tissue Bank, Department of Comparative Biomedicine and Food Science, University of Padova, viale dell’Università, 16, 35020 Legnaro – Agripolis PD, Italy.

## Author Contributions

CCe and GB performed all the experimental procedures, data analysis, and contributed equally to manuscript writing. GF participated in performing data analysis and manuscript writing for the sequence comparison and phylogenetic analysis. GP, CCa, AP, FG, FDN, DI, and MS gave contributions in the acquisition of data for the work and microbiological analysis. GDG. and SM supervised the study design, experimental procedure, data analysis, and manuscript writing. All authors reviewed and agreed on the current version of the manuscript.

## Conflict of Interest Statement

The authors declare that the research was conducted in the absence of any commercial or financial relationships that could be construed as a potential conflict of interest.

## Footnotes

1 http://www.dnastar.com/t-dnastar-lasergene.aspx

## Supplementary Material

FIGURE S1 **(A)** The two boxes represent the complete sequence of N gene (GenBank Provisional Acc. No. KX237510) and P/V/C gene (GenBank Proviasional Acc. No. KX237511) identified in Cuvier’s beaked whale’s lung tissue. **(B)** Agarose gel electrophoresis for detection of dolphin morbillivirus (DMV) RNA with nested PCR using primer pairs DMV-11 (614 bp) on the left and DMV-13 (200 bp) on the right (Centelleghe et al., 2016); PCR with DMV13 primer pair was performed with 614 bp amplicon as template. The box shows the detected 614 bp sequence representing partial H gene (GenBank Provisional Acc. No. KX237512); the 200 bp sequence is highlight in blue. M, DNA ladder 100 bp (Invitrogen); 1, positive control for DMV; 2, Cuvier’s beaked whale lung; NC, negative control.

FIGURE S2 **Multiple sequences alignment of seven DMV P gene partial sequences previously reported close related to that identified in *Ziphius Cavirostris* tissues (underlined in red), herein reported (provisional GenBank Acc. No. KX237511), generated with ClustalW2 program and shown together with their GenBank Accession Number.** Identical nucleotides are indicated by asterisks.
