# Supplementary figures and images for: Dolphin Morbillivirus in a Cuvier’s Beaked Whale (Ziphius cavirostris), Italy
Source: Front Microbiol. 2017 Jan 31;8:111. doi: 10.3389/fmicb.2017.00111 (PMC5281547; doi:10.3389/fmicb.2017.00111)

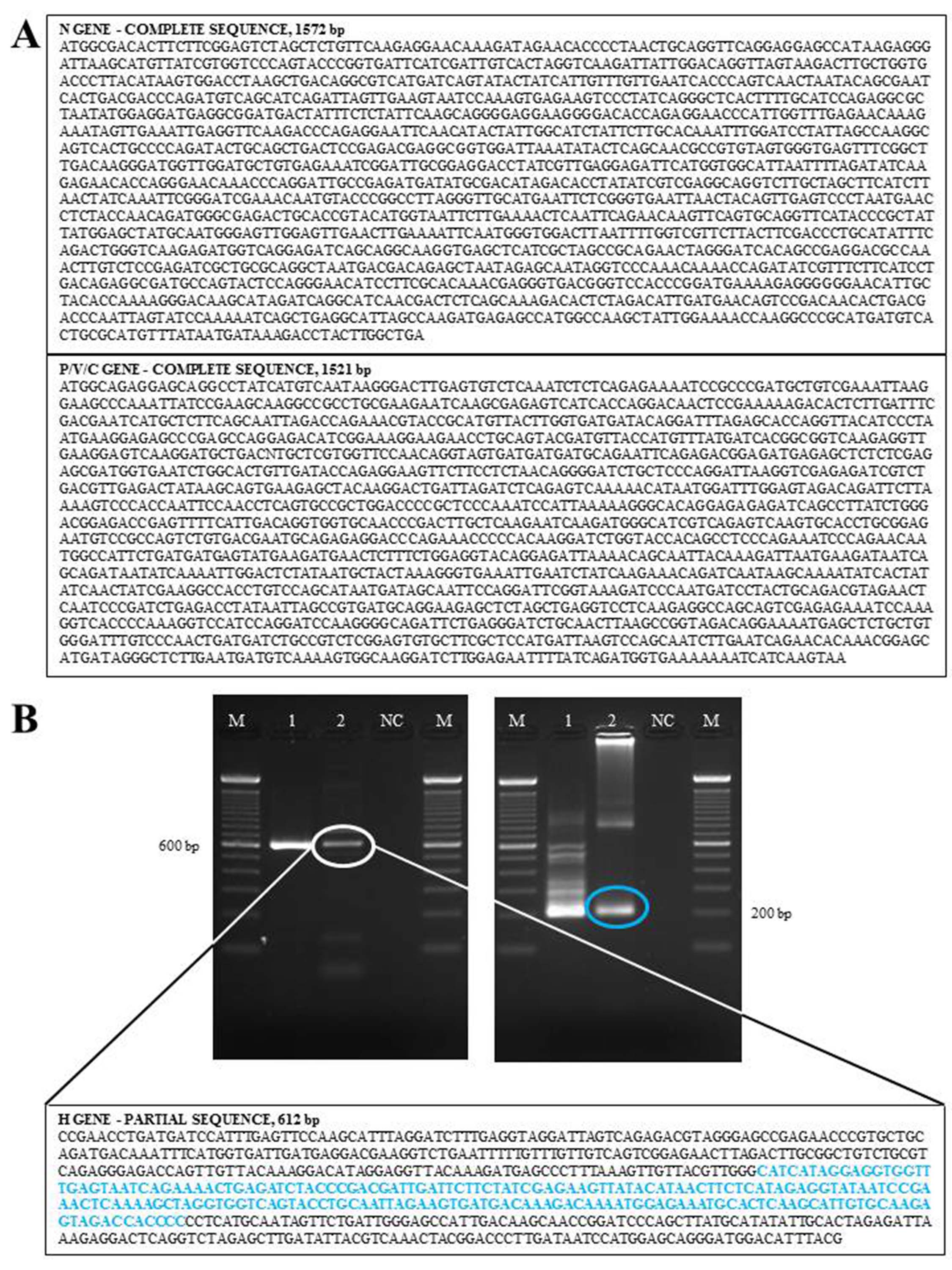

Supplement: FIGURE S1 — (A) The two boxes represent the complete sequence of N gene (GenBank Provisional Acc. No. KX237510) and P/V/C gene (GenBank Proviasional Acc. No. KX237511) identified in Cuvier’s beaked whale’s lung tissue. (B) Agarose gel electrophoresis for detection of dolphin morbillivirus (DMV) RNA with nested PCR using primer pairs DMV-11 (614 bp) on the left and DMV-13 (200 bp) on the right (Centelleghe et al., 2016); PCR with DMV13 primer pair was performed with 614 bp amplicon as template. The box shows the detected 614 bp sequence representing partial H gene (GenBank Provisional Acc. No. KX237512); the 200 bp sequence is highlight in blue. M, DNA ladder 100 bp (Invitrogen); 1, positive control for DMV; 2, Cuvier’s beaked whale lung; NC, negative control. [file Image_1.JPEG]

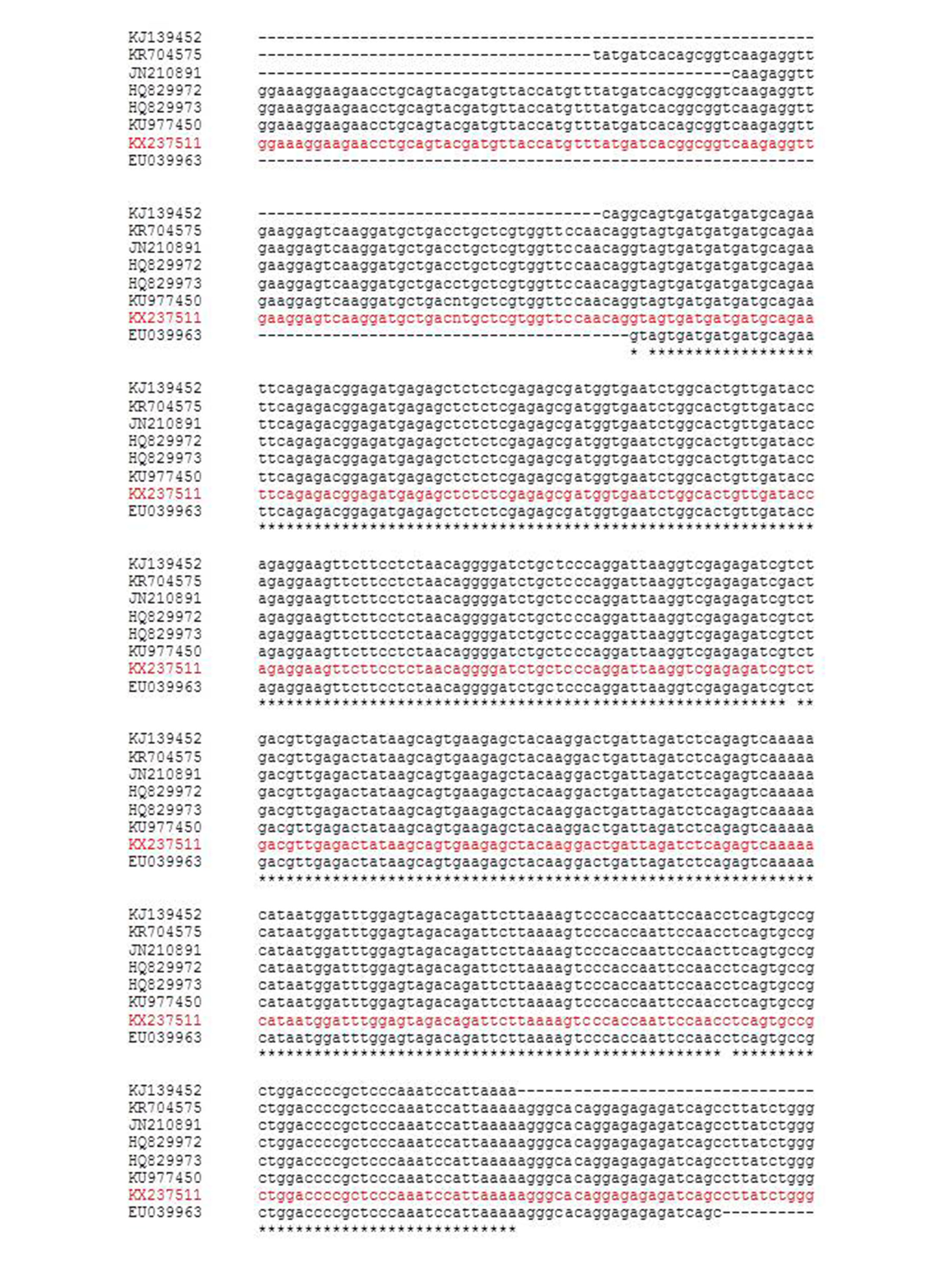

Supplement: FIGURE S2 — Multiple sequences alignment of seven DMV P gene partial sequences previously reported close related to that identified in Ziphius Cavirostris tissues (underlined in red), herein reported (provisional GenBank Acc. No. KX237511), generated with ClustalW2 program and shown together with their GenBank Accession Number. Identical nucleotides are indicated by asterisks. [file Image_2.JPEG]
